# Decomposition analysis of public health service utilization and health disparities among urban and rural older adult migrants in China

**DOI:** 10.3389/fpubh.2025.1591804

**Published:** 2025-06-18

**Authors:** Bo Dong, MengHan Jiang, Jing Zong

**Affiliations:** ^1^School Public Health, Zhejiang Chinese Medicine University, Hangzhou, China; ^2^School of Humanities and Management, Zhejiang Chinese Medical University, Hangzhou, China; ^3^The 960th Hospital of the PLA Joint Logistics Support Force, Jinan, Shandong, China

**Keywords:** China, older adult migrant population, public health equalization, health equity, urban–rural differences

## Abstract

**Background:**

In the context of China’s aging population and increasing internal migration, “old age” and “mobility” are the special dual attributes of the older adult migrants, and their utilization of basic public health services and health protection is also a major public health issue. Against the backdrop of growing urban–rural development disparities in China, this study aims to examine the differences in public health service utilization and health status between urban and rural older adult migrants and quantify the contribution of relevant influencing factors.

**Methods:**

This study utilized data from the 2018 China Migrants Dynamic Survey (CMDS). After data cleaning, a final sample size of 4,198 participants was analyzed. Descriptive analysis and chi-square tests were used to examine the distribution of three types of public health services—health education, health record establishment, and family doctor contracting—as well as self-reported health status among urban and rural older adult migrants. Multiple linear regression models were applied to identify factors associated with public health service utilization and health status. Finally, the Blinder–Oaxaca decomposition method was used to quantify the extent to which various factors contributed to urban–rural disparities in health service utilization and health outcomes.

**Results:**

Rural older adult migrants exhibited slightly higher utilization rates of public health services—health education, health records, and family doctor contracting—compared to their urban counterparts. However, their self-reported health status was significantly lower (76.88% vs. 79.12%). Regression analysis revealed that age, mobility range, education level, income, health insurance coverage, and geographic region were significant factors influencing both service utilization and health outcomes. Public health service use was positively associated with better health in both urban and rural groups. The Blinder–Oaxaca decomposition indicated that age, mobility range, and household income were the primary contributors to urban–rural disparities in health education; age, mobility range, and region contributed most to differences in health record establishment and family doctor contracting; and family doctor contracting, age, mobility range, and region were key drivers of disparities in health status.

**Conclusion:**

There are differences in public health service utilization and health status between urban and rural older adult migrants in China. While rural older adult migrants use public health services at a slightly higher rate, they experience worse health outcomes than their urban counterparts—largely due to socio-economic and regional disparities. Targeted interventions aimed at improving access, enhancing health education, optimizing service delivery, and strengthening policy support are essential to narrowing these urban–rural health gaps and promoting health equity among older adult migrants.

## 1 Introduction

In China, internal migrants are individuals whose current residence differs from their household registration, also known as Hukou (1). This population has made significant contributions to China’s rapid economic development (2), and often serves as a crucial survival strategy for millions of rural residents (3). China has the world’s largest internal migrant population. According to the 2021 Seventh National Census, the number of internal migrants reached nearly 380 million in 2020—an increase of 150 million (70%) since 2010 (3). As China’s society and economy continue to develop, large-scale population mobility is expected to persist. Within this migrant population, older adult migrants constitute a distinct subgroup. Studies indicate that the number of older adult internal migrants grew rapidly after 2000, reaching 13.04 million—5.3% of the total migrant population—by 2015 (4). This population is projected to increase further, driven by factors such as population aging, family reunification, caregiving responsibilities, and the pursuit of improved living conditions.

“Old age” and “mobility” are dual defining characteristics of older adult migrants. Older adults are at increased risk of illness due to age-related physical frailty, making their health status a critical concern (5). As individuals age, they experience immune senescence—a gradual decline in immune system function—which renders them more susceptible to infections and other diseases. Additionally, many older adults suffer from multiple chronic conditions, such as hypertension and diabetes, which may interact and compound overall health risks. At the same time, mobility status plays a key role in limiting access to public services (6). Mobility status refers to the migrant identity of individuals, which often functions as a barrier to the effective utilization of public health services. As migrants, older adult individuals frequently face challenges in accessing systematic healthcare and generally experience lower levels of service availability and accessibility (7). Therefore, given the combined influence of aging and mobility, it is essential to pay special attention to both the health status of older adult migrants and their utilization of public health services.

The urban–rural dualistic structure has long been a defining feature of China’s social and economic system. This structure is characterized by marked disparities between urban and rural areas in social, economic, and institutional dimensions (8, 9). Its core manifestation lies in persistent inequalities in economic development, access to social welfare, and political rights. These disparities have had a lasting impact on the quality of life of both urban and rural residents and have significantly influenced patterns of social mobility. At the heart of this dual structure is the household registration system, known as Hukou. Under this system, individuals’ access to social welfare, education, healthcare, employment opportunities, and other benefits is closely tied to their place of official registration (10, 11). Urban residents usually enjoy better social security, educational resources, and medical services, whereas rural residents are often in a more disadvantaged position. Migrant populations, especially those from rural areas, face significant challenges in accessing social security and public services when compared to their urban counterparts. Although the Chinese government has, in recent years, introduced a range of reforms aimed at narrowing the urban–rural divide—such as promoting rural development and enhancing urban–rural integration—substantial gaps between urban and rural areas persist.

The Anderson Behavioral Model of Health Service Utilization is widely used in health services research to analyze the relationships among contextual factors, individual characteristics, health behaviors, and health outcomes (12–14). The model offers a comprehensive, multidimensional framework that effectively captures the various determinants influencing health service utilization across different population groups. In the Chinese context, notable disparities in economic and social development persist between urban and rural areas (15, 16). These disparities are evident not only in the distribution of material resources but also in aspects such as social structure, quality of life, social security, education, and healthcare. Urban and rural older adult migrants experience distinct living conditions, and these differences significantly shape their health service utilization patterns and health behavior choices, thereby contributing to variations in health outcomes. For instance, studies have shown that the proportion of urban migrants participating in health insurance in their destination areas is significantly higher than that of rural migrants (54.07% versus 45.93%). Participation in local health insurance schemes influences health outcomes by affecting individuals’ likelihood of utilizing public health and medical services (17).

To improve public health access and health equity for migrants, China launched the Migrant Health Equalization Program in 2013. This program aims to improve the equalization of public health services for the floating population by implementing health education initiatives, establishing and maintaining health records, and improving access to medical services. In 2016, China issued the “Healthy China 2030 Planning Outline,” which explicitly aims to “promote the equalization of basic public services in the field of health” and to “gradually reduce the differences in basic health services and health levels between urban and rural areas, regions, and population groups.” Although the policy of public health service equalization is still being advanced incrementally, it reflects a long-term national commitment to reducing health inequalities. In recent years, scholars have paid increasing attention to issues surrounding public health service utilization and the health outcomes of the migrant population. Existing research has examined the current status and equity of public health service use among migrants, as well as the impact of service utilization on health outcomes. However, there remains a gap in the literature regarding the older adult migrant population—a unique and vulnerable group—particularly in analyses that account for urban–rural disparities. In light of this, the present study focuses specifically on older adult migrants, adopting a micro-level perspective to explore differences between urban and rural populations. First, it examines disparities in the utilization of public health services and health outcomes between urban and rural older adult migrants. Second, it employs regression analysis to identify factors influencing public health service utilization and health in both groups. Finally, it applies the Blinder–Oaxaca decomposition method to quantify the extent to which these factors contribute to differences in public health service utilization and health outcomes between urban and rural older adult migrants. The objectives of this study are threefold: (1) to describe the current differences in public health service utilization and health outcomes between urban and rural older adult migrants; (2) to analyze the differences in influencing factors affecting public health service utilization and health in these two groups; and (3) to quantitatively assess the contribution of each factor to the observed disparities. To achieve these objectives, the study utilizes data from the China Migrants Dynamic Survey (CMDS), which provides extensive and representative data on China’s migrant population. The CMDS includes detailed information on public health service use, health status, income, household registration, mobility patterns, and reasons for migration. Its comprehensive scope and high reliability make it a valuable resource for analyzing the health and healthcare utilization patterns of migrant groups in China (18–20).

Compared with existing studies, this research makes three key contributions. First, it utilizes large-scale micro-survey data to provide a comprehensive, multidimensional, and multilevel analysis of public health service utilization and health outcomes among older adult migrants. This approach offers a holistic understanding of the health status of this population. Second, the study systematically examines the differences in public health service utilization and health status between urban and rural older adult migrants, while further exploring the divergent influencing factors between the two groups. This analysis contributes valuable empirical evidence to support the refinement of public health and health protection policies for migrant populations. Third, the study employs a decomposition approach to analyze the extent to which various factors contribute to disparities in public health service utilization and health outcomes between urban and rural older adult migrants. This enables the identification of key drivers of inequality, thereby supporting more targeted and effective policy interventions to promote the equalization of public health services and improve health equity across urban and rural older adult migrant groups.

## 2 Literature review

Compared to other populations, migrants represent a vulnerable and underserved demographic. Research across different countries consistently highlights that the majority of migrants have low levels of education, are predominantly employed in labor-intensive occupations, and experience poor working and living conditions. These factors contribute to an increased vulnerability to health risks (21, 22). Additionally, migrants often lack the same social support and protection as residents, and their access to public health services is typically more limited (23, 24). As a result, the utilization of public health services and health protection for migrant populations has garnered significant attention from scholars.

The utilization of public health services and the health outcomes of the migrant population have been widely studied, and the findings can be summarized into four key areas. First, studies consistently show that the utilization of public health services among migrants is lower than that of local residents. For instance, Aihua et al. reported that only 30.37% of migrants in the Pearl River Delta accessed basic public health services, compared to 43.23% of local residents (25). Similarly, Sun et al. conducted a study on community health service access in Shanghai and found that migrants had lower participation rates in health education and community clinic visits compared to local residents. Additionally, nearly half of migrants were unaware of public health service equalization policies (26). Second, studies focusing on specific migrant subgroups, such as those with chronic diseases, have highlighted disparities in health service utilization. For example, Yan et al. found that only 39.4% of migrant individuals with chronic diseases accessed community health services, compared to 65.6% of local residents. Moreover, 36.4% of migrant patients received chronic disease education, compared to 55.5% of local residents (27). Research by Gu et al. on China’s ethnic minority migrant population revealed similarly low rates of basic public health service utilization, with only 81.1% receiving health education, 29.2% having health records, and 12.2% enrolled in family doctor services (28). Third, studies examining the younger migrant population have shown that the level of basic public health service utilization is particularly low among this group. Qiong et al. found that only 18.0% of migrants aged 15–24 years had established health records, and 28.5% had received health education on occupational diseases. Additionally, 43.0 and 22.6% of the same group had received health education on the prevention and treatment of HIV/AIDS and tuberculosis, respectively (27). Furthermore, 38.4 and 31.6% of these individuals had received health education on reproduction and contraception and sexually transmitted diseases, respectively. Furthermore, 38.4 and 31.6% of these individuals had received health education on reproduction and contraception and sexually transmitted diseases, respectively (29). Regarding health outcomes, existing studies have shown that the health status of the migrant population is also significantly lower than that of the household population, underscoring the need to strengthen health protection to improve the overall health status of migrant populations (30–32). When it comes to the older adult migrant population, studies have largely focused on their receipt of health education, health records, and family doctor contracts. These studies have revealed that the older adult migrant population’s utilization of basic public health services remains low (33–35), and their health status is often poor, with their health issues frequently not receiving adequate attention (36).

Regarding the influencing factors of public health service utilization and the health of migrant populations, research shows that marriage, education, hukou (household registration), migration distance, and medical insurance significantly affect the utilization of basic public health services among older adult migrants (37–39). In terms of health, in addition to the factors mentioned above, the older adult migrant population’s own health education, health records, and behaviors related to public health service utilization also impact their health (40, 41).

Urban–rural disparities in public health service utilization and health among migrants remain underexplored. Most existing studies focus on the differences between migrant and local household populations or examine equity in service utilization and health within the migrant population itself (42, 43). However, there is limited analysis specifically addressing the disparities between urban and rural older adult migrants. For instance, Wang et al. analyzed disparities in the knowledge and utilization of basic public services between migrants and household residents, finding that migrants continue to face substantial gaps in both awareness and use of national public health services compared to the household population (44). Deng et al. reported that family doctor contracting services were more frequently utilized by migrants with higher incomes, and that factors such as age, gender, household registration, literacy, economic status, and service accessibility significantly influenced service utilization (45). Similarly, Liu et al. found that the concentration index for self-assessed health among migrants was 0.0217 overall—0.0216 for migrants from urban households and 0.0219 for those from rural households—indicating a health equity bias favoring higher-income groups, with rural migrants experiencing greater health disparities than their urban counterparts (46).

In addition to disparities, the health effects of public health service utilization among migrants have also received scholarly attention. Existing studies have demonstrated that access to basic public health services significantly contributes to improved health outcomes among the migrant population. For example, Cheng et al., using data from the 2017 CMDS, found that the equalization of public health services had a positive impact on the health status of migrants (47). Similarly, the study by Xu et al. affirmed that the implementation of basic public health services in China plays a crucial role in promoting the health of the floating population (48).

The reviewed studies demonstrate that scholars have paid considerable attention to the utilization of public health services and health outcomes among older adults who migrate, providing a solid foundation for the present research. However, several gaps remain that warrant further investigation. First, existing research has a narrowly focused scope. Insufficient emphasis has been placed on older adults with migrant status as a distinct group with unique characteristics in terms of public service utilization and health outcomes. Given China’s deepening aging population and the national emphasis on healthy aging, there is a pressing need to further examine and prioritize the public health and wellbeing of this demographic. Second, few studies adopt a contextual perspective on urban–rural disparities. The dichotomous urban–rural structure in China necessitates attention to these contextual differences, yet research comparing public health service utilization and health outcomes among urban versus rural older migrants remains scarce. Finally, the scope of existing research requires enrichment. Most studies focus on the utilization or equity of individual public health services, lacking a comprehensive perspective that considers the equitable access to multiple services and the relationship between such utilization and overall health outcomes in this population.

## 3 Methods

### 3.1 Data source

This study utilized data from the 2018 China Migrants Dynamic Survey (CMDS), a nationally representative cross-sectional survey of individuals with migrant status conducted annually by the National Health Commission of China since 2009 (49). The 2018 dataset, the most recent available at the time of this study, reflects contemporary demographic trends, patterns in public health service utilization, and health outcomes among the domestic migrant population. Its timeliness enhances the relevance of the findings. Moreover, the 2018 survey featured an expanded sample size and broader regional coverage, thereby including a wider range of groups—particularly those previously underrepresented. The content of the 2018 CMDS was also updated, with newly added questions related to public health services, health status assessments, and social security access. These improvements aimed to better capture the health needs and challenges of individuals with migrant status.

The CMDS is recognized for its high representativeness and low sampling error (50). The survey employed a stratified, multi-stage, probability-proportional-to-size (PPS) sampling method (51). In this case, the stratification was done as follows: first, the 32 provincial-level administrative regions in China were taken as subpopulations, and within the subpopulations, stratification was done based on provincial capital cities, planned cities, and key cities or regions. Second, provincial capital cities, separately listed cities, and individual key cities or regions were treated as mandatory layers, and other cities were treated as one layer. In addition, implicit stratification was performed within each layer by sorting by city administrative division and township (township, street) attributes. Finally, within the sampled townships (towns and streets), stratification was performed based on village (or neighborhood) committees and the residence patterns of individuals with migrant status. The CMDS employed a four-stage, stratified, multi-stage probability-proportional-to-size (PPS) sampling approach to ensure sample representativeness. In the first stage, at the national level, regions with high concentrations of individuals with migrant status were identified as primary sampling units. These areas spanned all provincial-level administrative divisions, providing broad geographic and demographic representation. In the second stage, villages or neighborhood committees within each sample point were selected using the PPS method, meaning that locations with larger migrant populations had a greater probability of selection. The third stage involved household sampling. Within each selected village or neighborhood committee, lists of eligible households were compiled, and a random sample was drawn. In the final stage, individual-level surveys were conducted. Trained interviewers administered face-to-face questionnaires to each eligible household member with migrant status. This rigorous sampling design ensures both statistical reliability and comprehensive coverage of the target population.

The survey also adopted rigorous methods to ensure data quality, including the scientific design of the questionnaire, the training of enumerators, the setting up of survey supervisors to verify the questionnaire, and the quality checking by means of telephone callbacks. The questionnaire design process was grounded in a clearly defined indicator system aligned with the objectives of the survey. Using the questionnaire management subsystem of the integrated survey platform, the research team developed and refined the questionnaire through expert consultations and pilot testing to ensure its relevance and clarity. Enumerator training was standardized through a unified training manual and the training management subsystem of the integrated survey platform. Initial training was followed by examinations; only those who passed were allowed to participate in the household survey. Secondary training was also conducted to reinforce skills and ensure consistency. All investigators must pass the examination before they can enter the household survey. Questionnaire verification involved selecting grassroots-level personnel as both supervisors and investigators. These individuals underwent centralized training to ensure a consistent understanding of survey content and methodology. Investigators were responsible for conducting interviews and performing initial checks on completed questionnaires to identify omissions or inconsistencies. Supervisors then conducted a secondary review of the forms. For quality assurance, the survey enhanced its data collection system with logical validation and early warning features. It also leveraged the implementation management subsystem and a dedicated telephone survey system to perform logical checks and telephone callbacks as part of the data verification process. Additionally, technical guidance and oversight were strengthened through real-time review of completed questionnaires, random sampling, and in-person verification during field visits. These procedures collectively ensured the reliability and integrity of the data collected.

The CMDS targeted individuals aged 15 years and above from 31 provinces and the Xinjiang Production and Construction Corps who had resided in their current location for more than 1 month and were not registered as local household members (i.e., not part of the district, county, or city household registry). The 2018 CMDS collected data from 152,000 respondents, covering a wide range of topics, including basic demographic information, mobility patterns and intentions, employment and social security, income and expenditure, housing conditions, access to basic public health services, marriage and family planning services, children’s mobility and education, as well as cultural and psychological aspects. This breadth of content provides a rich set of variables for analysis. Given the focus of this study on the older adult migrant population, respondents younger than 60 years were excluded (*N* = 145,770). Further, cases with missing values or outliers were removed (*N* = 2,032), resulting in a final analytic sample of 4,198 older adult migrant individuals. Compared with existing studies on public health service utilization and health outcomes among the migrant population (52, 53), the data processing procedures adopted in this study align with standard practices. Moreover, the sample size of older adult migrants in this study is relatively larger than that of comparable research (54, 55). The rigorous data processing steps and adequate sample size provide a robust foundation for investigating public health service utilization and health disparities among older adult migrants, and they strongly support the research objectives of the present study.

### 3.2 Variables

#### 3.2.1 Outcome variables

The outcome variables of interest in this study are public health service utilization and the health of the older adult migrant population. In the existing literature, there is some variation in how public health service utilization is measured among migrant populations. For example, some studies have considered indicators such as health education, health records, free contraceptive distribution, and free contraceptive surgical services as dimensions of public health service utilization (56). Other studies have limited their focus to health education and the establishment of health records (57). Additionally, Min Shuhui et al. included three components—health education, health records, and family doctor contracting—to assess older adult migrants’ utilization of public health services (58). Based on relevant studies (20, 59) and considering the availability of data, this study adopts three indicators to measure public health service utilization among the older adult migrant population: health education, health records, and family doctor contracting. Specifically, health education was assessed based on the question: “During the past year, have you received health education in the following areas at your current place of residence or organization?” This includes seven categories of health education, such as prevention and treatment of occupational and chronic diseases. Respondents who received at least one form of health education are coded as 1, and those who did not as 0. Health records were measured using the question “Have you set up a health record of your residents in your local area?” with responses coded as 1 for “yes” and 0 for “no.” Similarly, family doctor contracting is measured by the question: “Have you contracted with a local family doctor?” with “yes” coded as 1 and “no” as 0.

Self-assessed health was used to measure the health status of the migrants population. Self-assessed health status has been reported to be consistent with the actual health level of the individual (60, 61), and it serves as an indicator that can be applied across various contexts and can be used as a proxy for actual health status (62). In this study, health status was assessed with the question: “How is your health?” Respondents could choose from four options: not able to take care of yourself; unhealthy, but able to take care of yourself; basically healthy; and healthy. These responses were categorized into three levels to facilitate analysis: 1 = unhealthy (including ‘unable to self-care’ and ‘unhealthy but independent’), 2 = basically healthy, 3 = healthy. Thus, self-rated health was treated as an ordinal variable in this study, with higher values indicating better health status.

#### 3.2.2 Grouping variables

The hukou system, established in the late 1950s, assigned individuals a household registration based on their birthplace (63, 64). In the early stage (1950s–1980s) the system was primarily designed to restrict internal population mobility and functioned as a key mechanism for resource allocation under the planned economy (65), especially reinforcing the dual urban–rural social welfare structure (66). Since the introduction of market-oriented reforms in the 1980s, the hukou system has undergone significant changes to accommodate the growing demand for urban labor. The Chinese government’s stance on internal migration has gradually shifted from one of “restriction” to “encouragement and assistance” (21), migrants no longer face existential challenges solely due to lacking hukou status (67). However, the hukou system continues to hinder internal migrants from fully accessing the benefits of China’s economic and social development (68). On the one hand, *hukou* status remains closely tied to access to public welfare benefits. On the other hand, the process of changing hukou status remains highly restrictive for internal migrants (63, 64).

Given the urban–rural dichotomy in China, this study classified respondents as either urban or rural based on their hukou location. This classification aims to capture the structural disparities between urban and rural areas in terms of economic development, the allocation of public health service resources, and overall population health status. By doing so, the analysis is better positioned to reflect and examine the contextual differences that shape public health service utilization and health outcomes among older adult migrants across urban and rural settings.

#### 3.2.3 Covariates

This study is based on Anderson Behavioral Model of Health Service Utilization (69, 70) and incorporates covariates selected based on data availability. These covariates, used to adjust for confounding effects, are categorized into three domains: predisposing characteristics; enabling resources; and contextual factors, enabling resources, and situational characteristics. Predisposing characteristics include gender, age, marital status, and educational attainment (20). Enabling resources include household income level, employment status, and health insurance (71); and situational characteristics include migration scope and reasons for mobility (49). Based on the available data, the variables were operationalized as follows: gender was coded as a binary variable (male = 1, female = 0); age was calculated as the difference between the year of interview and the year of birth; marital status was a dummy variable (first married, remarried, or cohabiting = 1, unmarried, divorced, or widowed = 0); educational attainment was measured on a scale of 0–4 (never been to school = 0, elementary school = 1, junior high school = 2, senior high school = 3, and university and above = 4); household income was categorized into provincial income percentiles (<20th, 20th–39th, 40th–59th, 60th–79th, ≥80th); employment status was coded as a binary variable (unemployed = 0, employed = 1); health insurance was categorized as Basic Medical Insurance for Urban and Rural Residents (BMISURR,1) or Urban Employee Basic Medical Insurance (UEBMI, 2); migration scope was coded as intercounty = 1, intercity = 2, or interprovincial = 3; and reason for mobility was coded as mobility for work = 1 and mobility for other reasons = 2. Given the varying economic and social environments across provinces, which may influence the utilization of public health services and the health outcomes of the migrant population, this study categorizes the provinces into three geographic regions: eastern (value = 3), central (value = 2), and western (value = 1) China. This regional classification enables a more nuanced analysis of spatial disparities in health service access and health status among older adult migrants. In combination with the aforementioned variables, the definitions and codings of all variables included in this study are presented in Table 1.

**Table 1:** Definition of variables.

Variables			Definition
Outcome variables	Public health services utilization	Health education	Not accepted = 0; Accepted = 1
		Health record	Not established = 0; Established = 1
		Family doctor contract	Not contracted = 0; Contracted = 1
	Health		Unhealthy = 1; Basically healthy = 2; Healthy = 3
Grouping variables	Place of household registration		Rural; Urban
Covariates	Predisposing characteristics	Gender	Male = 1; Female = 2
		Age	60–70 = 1; 71–80 = 2; 81 + = 3
		Educational level	Illiterate = 1; Elementary school = 2; Middle school = 3; High school = 4; University/college = 5
		Marriage	Unmarried = 0; Married = 1
	Enabling resources, and	Work	Not worked = 0; worked = 1
		Income	Lowest (<20th percentile) = 1
			Lower (20–39th percentile) = 2
			Middle (40–59th percentile) = 3
			Higher (60–79th percentile) = 4
			Highest (≥80th percentile) = 5
		Health insurance	BMISURR = 1; UEBMI = 2
	Contextual factors	Mobility range	Intercounty = 1; Intercity = 2; Interprovince = 3
		Reasons for mobility	Family = 1; Work = 2; Other = 3
		Region	West = 1; Central = 2; East = 3

### 3.3 Statistical models

#### 3.3.1 Binary logistic regression model

To analyze the factors influencing the utilization of public health services among urban and rural older adult migrant populations, this study employs a binary logistic regression model, given that the three dependent variables—health education, health records, and family doctor contracting—are all binary outcomes. The model specification is as follows:

$$\text{logit}=\frac{P_{i}}{1-p_{i}}=\beta_{0}+\beta_{1}X_{i}+\varepsilon_{i}$$

Where $P_{i}$ denotes the probability of public health service utilization, $\beta_{0}$ is the intercept term, X_i_ represents covariates (predisposing, enabling, and contextual factors), including three types of factors, namely, propensity characteristics, situational characteristics, and enabling resources, $\beta_{1}$ denotes the influence coefficients of the relevant factors on the utilization of public health services for the older adult, and $\varepsilon_{i}$ is the error term.

#### 3.3.2 Ordered multicategorical logistic regression model

To analyze the factors influencing the health of urban and rural older adult migrant populations, this study employs an ordered logit model, as the dependent variable—self-assessed health status—is an ordinal variable with three categories (1 = unhealthy, 2 = basically healthy, 3 = healthy). The model is specified as follows:

$$\text{logit}(\frac{P(Y\leq j)}{1-P(Y\leq j)})=\beta_{0}+\beta_{1}X_{i}+\varepsilon_{i}$$

Where *Y* represents the self-assessed health status of the migrants, $\beta_{0}$ is the intercept term, X_i_ represents other factors affecting the health of the older adult, $\beta_{0}$ represents the coefficient of influence of the relevant factors on health, and *ε* is the error term.

#### 3.3.3 Blinder–Oaxaca decomposition analysis

To quantify the urban–rural disparities in public health service utilization and health outcomes among older adult migrants, this study employed the Blinder–Oaxaca decomposition technique to disaggregate the contributions of various determinants. The Blinder–Oaxaca methodology enables the decomposition of mean outcome differences into two components: the explained component, which is attributable to differences in observable characteristics between groups, and the unexplained component, which reflects differences arising from unobserved heterogeneity or unequal returns to these characteristics (72). This analytical approach thus partitions the observed disparities into two primary components: explained variations (arising from group differences in measured characteristics) and unexplained variations (attributable to differential returns to these characteristics) (72, 73). The model used is as follows:

$${\bar{y}}_{a}-{\bar{y}}_{b}=({\bar{x}}_{a}-{\bar{x}}_{b})\beta_{b}+{\bar{x}}_{b}(\beta_{a}-\beta_{b})$$

where a and b represent the groups of older adult migrant population in urban and rural regions, ${\bar{y}}_{a}$ and ${\bar{y}}_{b}$ represent the utilization of public health services and health status of different groups respectively, $({\bar{x}}_{a}-{\bar{x}}_{b})\beta_{b}$ is the “explained component,” i.e., differences in public health service utilization or health due to variations in characteristics across groups; ${\bar{x}}_{b}(\beta_{a}-\beta_{b})$ is the “unexplained component,” which can be interpreted as the differences caused by unobservable factors other than those already considered in the model. By quantifying these components, the method provides insights into the extent to which disparities are driven by structural inequalities versus differential returns to observed attributes (74, 75).

### 3.4 Data analysis

**Figure 1:**
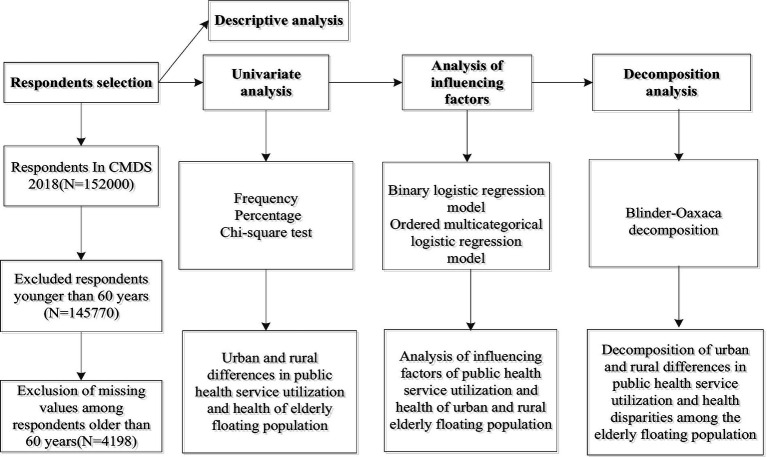
The flow chart of the study.

Descriptive statistics and chi-square tests were used to characterize respondents and assess urban–rural disparities in public health service utilization and health outcomes among older adult migrants. Logistic regression models were employed to analyze determinants of public health service utilization (binary outcomes) and self-rated health (ordinal outcome). All analyses were conducted using SPSS 21.0. Blinder–Oaxaca decomposition was performed using Stata MP 16.0 to quantify factors contributing to urban–rural disparities. *p* < 0.05 was considered statistically significant. The study flowchart is presented in Figure 1.

## 4 Results

### 4.1 Participant’ characteristics

Table 2 presents urban–rural differences in public health service utilization and health outcomes among the 4,198 included respondents, of whom 58.93% were rural migrants and 41.07% were urban migrants. Rural migrants exhibited slightly higher utilization rates for health education, health records, and family doctor contracting, with statistically significant differences in all three services. Conversely, urban migrants reported significantly better self-rated health (79.12% vs. 76.88%). Table 3 shows chi-square test results for 10 covariates, revealing significant urban–rural differences in seven variables (age, educational attainment, employment status, migration scope, income, health insurance, and region), whereas gender, marital status, and migration motives did not differ between groups.

**Table 2:** Urban–rural differences in utilization of public health services and health of the older adult migrant population.

Variables		Urban	Rural	*χ* ^2^	*p* values
		*N* (%)			
Health education	Not accepted	452 (26.22)	583 (25.57)	3.849	0.050
	Accepted	1,272 (73.78)	1,891 (76.43)		
Health record	Not established	1,239 (71.87)	1,713 (69.24)	3.361	0.067
	Established	485 (28.13)	761 (30.76)		
Family doctor contract	Not contracted	1,515 (87.88)	2,081 (84.11)	11.707	0.001
	Contracted	209 (12.12)	393 (15.89)		
Health	Unhealthy	139 (8.06)	283 (11.14)	13.258	0.001
	Basically healthy	211 (12.82)	289 (11.68)		
	Healthy	1,364 (79.12)	1,902 (76.88)		

**Table 3:** Sample characteristics of rural and urban respondents.

Variables		Total		Urban		Rural		*χ* ^2^	*p* values
		*n*	%	*n*	%	*n*	%		
Gender	Male	2,137	50.91	852	49.42	1,285	51.94	2.582	0.108
	Female	2,061	49.09	872	50.58	1,109	48.06		
Age	60–70	3,206	76.37	1,206	65.95	2,000	80.84	107.795	0.000
	71–80	780	18.58	448	25.99	332	13.42		
	≥81	212	5.05	70	4.06	142	5.74		
Education	Illiterate	106	2.53	25	1.45	81	3.27	33.8946	0.000
	Elementary school	565	13.46	141	8.18	424	17.14		
	Middle school	1,735	41.33	584	33.87	1,151	46.52		
	High school	895	21.32	385	22.33	510	20.61		
	University/college	897	21.37	595	34.26	308	12.45		
Marriage	Unmarried	662	15.77	286	16.59	376	15.20	1.481	0.224
	Married	3,536	84.23	1,438	83.41	2,098	84.80		
Work	Not worked	781	18.60	295	17.23	484	19.56	3.662	0.056
	worked	3,417	81.40	1,427	82.77	1,990	80.44		
Mobility range	Intercounty	698	16.63	225	13.05	473	19.12	73.278	0.000
	Intercity	1,261	30.04	445	25.81	816	32.98		
	Interprovince	2,239	53.33	1,054	61.14	1,185	47.90		
Reasons for mobility	Family	647	15.26	263	15.26	384	15.52	4.247	0.120
	Work	3,462	82.08	1,415	82.08	2,047	82.74		
	Other	89	2.67	46	2.67	43	1.74		
Income	Lowest (<20th percentile)	906	21.58	298	17.29	608	24.58	99.273	0.000
	Lower (20–39th percentile)	824	19.63	301	17.46	523	21.14		
	Middle (40–59th percentile)	892	21.25	351	20.36	541	21.87		
	Higher (60–79^t^h percentile)	806	19.20	348	20.19	458	18.51		
	Highest (≥80th percentile)	770	18.34	426	24.71	344	13.90		
Health insurance	BMISURR	2,896	68.99	1,002	58.12	1,894	76.56	161.392	0.000
	UEBMI	1,302	31.01	722	41.88	580	23.44		
Region	West	963	22.94	225	13.05	738	29.83	346.473	0.000
	Central	1,477	35.18	495	28.71	982	39.69		
	East	1,758	41.88	1,004	58.24	754	30.48		

### 4.2 Analysis of factors influencing the utilization of public health services and the health of urban and rural older adult migrants

#### 4.2.1 Health education

Table 4 presents the factors influencing the health education of the urban and rural older adult migrant population. Among urban older adult migrants, several characteristics were associated with a lower likelihood of receiving health education. These included being aged 71–80 years (odds ratio [OR] = 0.709, 95% confidence interval [CI]: 0.547–0.918), having interprovincial mobility (OR = 0.428, 95% CI: 0.289–0.634), belonging to the highest income group (OR = 0.630, 95% CI: 0.429–0.924), and residing in the central region (OR = 0.232, 95% CI: 0.149–0.359). In contrast, higher educational attainment was positively associated with receiving health education. Compared with those who had never attended school, the odds of receiving health education were higher among respondents with junior high school education (OR = 2.125, 95% CI: 0.894–5.055), senior high school education (OR = 2.573, 95% CI: 1.066–6.211), and university-level education (OR = 2.874, 95% CI: 1.178–7.013) i.e., there was a positive linear relationship between education level and health education Furthermore, urban older adult migrants enrolled in UEBMI were more likely to receive health education compared to those covered by the BMISURR (OR = 1.588, 95% CI: 1.203–2.095).

**Table 4:** Factors influencing health education among urban and rural older adult migrants.

Variables		Health education							
		Urban				Rural			
		*β*	OR	95%CI	*p*	*β*	OR	95%CI	*p*
Age	60–70 (Reference)								
	71–80	−0.344	0.709	−0.547 to 0.918	0.009	−0.085	0.918	0.698–1.208	0.542
	≥81	0.332	1.393	−0.779 to 2.493	0.264	0.062	1.064	0.702–1.613	0.770
Education	Illiterate (Reference)								
	Elementary school	0.644	1.904	0.757–4.789	0.171	−0.221	0.802	0.453–1.419	0.448
	Middle school	0.754	2.125	0.894–5.055	0.088	0.172	1.187	0.683–2.064	0.543
	High school	0.945	2.573	1.066–6.211	0.036	0.423	1.527	0.851–2.738	0.156
	University/college	1.056	2.874	1.178–7.013	0.020	0.360	1.433	0.722–2.658	0.254
Work	Not worked (Reference)								
	Worked	0.202	1.224	0.913–1.641	0.178	0.336	1.399	1.099–1.781	0.006
Mobility range	Intercounty (Reference)								
	Intercity	0.110	1.117	0.750–1.663	0.587	−0.144	0.866	0.651–1.151	0.322
	Interprovince	−0.849	0.428	−0.289 to 0.634	0.000	−0.310	0.733	0.548–0.981	0.037
Income	Lowest (<20th percentile) (Reference)								
	Lower (20–39th percentile)	−0.334	0.716	−0.490–1.047	0.085	0.146	1.157	0.873–1.534	0.311
	Middle (40–59th percentile)	−0.085	0.918	0.628–1.342	0.660	0.133	1.142	0.856–1.525	0.367
	Higher (60–79th percentile)	−0.273	0.761	0.516–1.122	0.168	−0.041	0.960	0.709–1.300	0.792
	Highest (≥80th percentile)	−0.462	0.630	−0.429 to 0.924	0.018	−0.228	0.796	0.572–1.108	0.176
Health insurance	BMISURR (Reference)								
	UEBMI	0.462	1.588	1.203–2.095	0.001	0.197	1.218	0.956–1.553	0.111
Region	West (Reference)								
	Central	−1.461	0.232	−0.149 to 0.359	0.000	−0.928	0.395	0.306–5.105	0.008
	East	−0.117	0.890	−0.575 to 1.378	0.601	−0.776	0.460	0.347–0.612	0.000
*R* ^2^		0.083				0.035			
*N*		1,724				2,474			

Among rural older adult migrants, reduced odds of receiving health education were observed among those who migrated across provinces (OR = 0.733, 95% CI: 0.548–0.981) and those residing in the central (OR = 0.395, 95% CI: 0.306–0.511) or eastern regions (OR = 0.460, 95% CI: 0.347–0.612). Conversely, employment was positively associated with health education uptake, with employed rural migrants having higher odds of participation compared to their unemployed counterparts (OR = 1.399, 95% CI: 1.099–1.781).

#### 4.2.2 Health records

Table 5 presents the factors affecting health record establishment among urban and rural older adult migrants. Among urban older adult migrants, a higher likelihood of health record establishment was significantly associated with having completed junior high school (OR = 4.048, 95% CI: 1.159–14.136), university education (OR = 4.025, 95% CI: 1.139–14.225), enrollment in UEBMI (OR = 1.535, 95% CI. 1.184–1.989), and residence in the East (OR = 5.114, 95% CI: 0.362–0.721). Conversely, the likelihood of establishing a health record was significantly lower among individuals aged 71–80 years (OR = 0.741, 95% CI: 0.563–0.976), those with intercity mobility (OR = 0.647, 95% CI: 0.462–0.905) or interprovincial mobility (OR = 0.403, 95% CI: 0.286–0.567), and those in the highest income bracket (OR = 0.712, 95% CI: 0.489–1.036).

**Table 5:** Factors influencing the health records of the urban and rural older adult migrant population.

Variables		Health records							
		Urban				Rural			
		*β*	OR	95%CI	*p*	*β*	OR	95%CI	*p*
Age	60–70 (Reference)								
	71–80	−0.299	0.741	0.563–0.976	0.033	−0.019	0.981	0.758–1.269	0.882
	≥81	−0.393	0.675	0.386–1.179	0.168	−0.223	0.800	0.546–1.171	0.251
Education	Illiterate (Reference)								
	Elementary school	1.072	2.921	0.801–10.651	0.104	−0.518	0.596	0.358–0.992	0.046
	Middle school	1.398	4.048	1.159–14.136	0.028	−0.234	0.791	0.488–1.283	0.343
	High school	1.054	2.869	0.814–10.114	0.101	−0.159	0.853	0.514–1.417	0.540
	University/college	1.392	4.025	1.139–14.225	0.031	−0.151	0.860	0.502–1.474	0.582
Work	Not worked (Reference)								
	Worked	−0.158	0.854	0.634–1.150	0.298	0.040	1.041	0.829–1.308	0.729
Mobility range	Intercounty (Reference)								
	Intercity	−0.435	0.647	0.462–0.905	0.011	0.003	1.003	0.789–1.274	0.983
	Interprovince	−0.909	0.403	0.286–0.567	0.000	−0.238	0.788	0.613–1.012	0.062
Income	Lowest (<20th percentile) (Reference)								
	Lower (20–39th percentile)	−0.123	0.884	0.608–1.284	0.517	0.001	1.001	0.771–1.300	0.992
	Middle (40–59th percentile)	−0.217	0.805	0.559–1.159	0.244	−0.073	0.929	0.713–1.210	0.586
	Higher (60–79th percentile)	−0.339	0.712	0.489–1.0357	0.076	−0.140	0.869	0.654–1.155	0.334
	Highest (≥80th percentile)	−0.223	0.800	0.554–1.154	0.232	−0.069	0.934	0.685–1.272	0.664
Health insurance	BMISURR (Reference)								
	UEBMI	0.428	1.535	1.184–1.989	0.001	0.432	1.540	1.241–1.912	0.000
Region	West (Reference)								
	Central	0.017	1.017	0.724–1.429	0.923	0.073	1.076	0.875–1.322	0.490
	East	1.632	5.114	0.362–0.721	0.000	−0.803	0.448	0.346–1.580	0.000
*R* ^2^		0.068				0.034			
*N*		1,724				2,474			

Among rural older adult migrants, a lower likelihood of health record establishment was associated with primary education (OR = 0.596, 95% CI: 0.358–0.922), interprovincial mobility (OR = 0.788, 95% CI: 0.613–1.012), and residence in the eastern region (OR = 0.488, 95% CI: 1.346–1.580). However, those enrolled in UEBMI were significantly more likely to have established health records compared to those covered by the BMISURR (OR = 1.540, 95% CI: 1.241–1.912).

#### 4.2.3 Family doctor contract

Table 6 presents the factors affecting family physician contract signing among urban and rural older adult migrants. Among urban older adult migrants, the likelihood of contracting with a family doctor was significantly lower among individuals aged 71–80 years (OR = 0.602, 95% CI: 0.394–9.199), those who were employed (OR = 0.673, 95% CI: 0.464–0.977), and those who had migrated across provinces (OR = 0.395, 95% CI: 0.254–0.614). Conversely, residence in the eastern region was associated with a significantly higher likelihood of signing a family physician contract compared to residence in the western region (OR = 4.260, 95% CI: 0.268–6.771).

**Table 6:** Factors influencing signing up for family doctors among the urban and rural older adult migrant population.

Variables		Family doctor contract							
		Urban				Rural			
		*β*	OR	95%CI	*p*	*β*	OR	95%CI	*p*
Age	60–70 (Reference)								
	71–80	−0.507	0.602	0.394–9.199	0.019	2.142	8.520	0.609–1.192	0.350
	≥81	−0.364	0.695	−0.337 to 1.431	0.323	2.134	8.450	0.532–1.342	0.476
Education	Illiterate (Reference)								
	Elementary school	0.960	2.613	0.553–12.334	0.225	1.986	7.289	0.395–1.344	0.311
	Middle school	0.775	2.171	1.481–9.791	0.313	2.095	8.124	0.453–1.457	0.486
	High school	0.932	2.540	0.558–11.553	0.228	2.273	9.708	0.526–1.791	0.924
	University/college	0.649	1.913	0.415–8.823	0.405	2.184	8.882	0.458–1.721	0.725
Work	Not worked (Reference)								
	Worked	−0.396	0.673	0.464–0.977	0.037	0.074	1.077	0.812–1.427	0.608
Mobility range	Intercounty (Reference)								
	Intercity	−0.271	0.762	0.512–1.136	0.182	−0.151	0.860	0.648–1.141	0.295
	Interprovince	−0.929	0.395	0.254–0.614	0.000	−0.385	0.681	0.502–0.922	0.013
Income	Lowest (<20th percentile) (Reference)								
	Lower (20–39th percentile)	−0.164	0.848	0.518–1.389	0.514	2.277	9.750	0.711–1.337	0.875
	Middle (40–59th percentile)	−0.338	0.713	0.435–1.1679	0.179	1.914	6.780	0.484–0.950	0.024
	Higher (60–79th percentile)	−0.291	0.747	−0.456 to 1.224	0.247	2.038	7.678	0.539–1.095	0.144
	Highest (≥80th percentile)	−0.265	0.767	0.471–1.251	0.288	1.850	6.361	0.426–0.949	0.027
Health insurance	BMISURR (Reference)								
	UEBMI	0.263	1.300	1.192–1.845	0.142	1.493	4.450	1.139–1.959	0.004
Region	West (Reference)								
	Central	0.110	1.117	0.738–1.691	0.602	0.100	1.106	0.863–1.417	0.428
	East	1.449	4.260	0.268–6.771	0.000	0.258	1.294	0.203–1.417	0.000
*R* ^2^		0.097				0.052			
*N*		1,724				2,474			

In rural areas, interprovincial migration was associated with lower odds of family doctor contracting (OR = 0.681, 95% CI: 0.502–0.922). Higher income quintiles (middle: OR = 0.678, 95% CI: 0.484–0.950; highest: OR = 0.636, 95% CI: 0.426–0.949) were linked to lower odds, while enrollment in UEBMI (OR = 4.450, 95% CI: 1.139–1.959) and eastern residence (OR = 1.294, 95% CI: 0.203–1.417) increased odds.

#### 4.2.4 Health

Table 7 presents the factors influencing self-rated health status among urban and rural older adult migrants. In urban areas, older adults who received health education had better health (OR = 1.315, 95% CI: 1.022–1.691), as did those with health records (OR = 1.706, 95% CI: 1.302–2.235) and family doctor contracts (OR = 1.149, 95% CI: 1.941–5.127). University education (OR = 2.583, 95% CI: 1.030–6.476), employment (OR = 1.726, 95% CI: 1.212–2.456), highest income quintile (OR = 1.758, 95% CI: 1.206–2.562), and eastern residence (OR = 1.293, 95% CI: 0.192–0.445) were also associated with improved health. Conversely, older age was negatively associated with health, as those aged 71–80 years (OR = 0.267, 95% CI: 0.208–0.383) and those over 80 years (OR = 0.383, 95% CI: 0.209–0.699) reported poorer self-rated health. Interprovincial migration was also linked to worse health outcomes (OR = 0.395, 95% CI: 0.258–0.603).

**Table 7:** Factors affecting the health of the urban and rural older adult migrant population.

Variables		Urban				Rural			
		*β*	OR	95%CI	*p*	*β*	OR	95%CI	*p*
Health education	Not accepted (Reference)								
	Accepted	0.273	1.315	1.022–1.691	0.033	0.348	1.416	1.073–1.864	0.013
Health record	Not established (Reference)								
	Established	0.534	1.706	1.302–2.235	0.000	0.379	1.462	1.098–1.946	0.009
Family doctor contract	Not contracted (Reference)								
	Contracted	1.149	3.155	1.941–5.127	0.000	0.975	2.652	1.674–4.200	0.000
Age	60–70 (Reference)								
	71–80	0.267		−0.208 to 0.343	0.000	−0.032	0.968	0.667–1.405	0.864
	≥81	0.383		−0.209 to 0.699	0.002	0.123	1.131	0.640–1.998	0.671
Education	Illiterate (Reference)								
	Elementary school	1.846		0.710–4.801	0.209	0.264	1.302	0.699–2.427	0.406
	Middle school	1.532		0.625–3.755	0.352	1.185	3.270	1.772–6.037	0.088
	High school	1.540		0.619–3.826	0.247	1.667	5.295	2.658–10.546	0.000
	University/college	2.583		1.030–6.476	0.043	1.730	5.642	2.622–12.137	0.000
Work	Not worked (Reference)								
	Worked	1.726		1.212–2.456	0.002	1.332	3.790	2.702–5.316	0.000
Mobility range	Intercounty (Reference)								
	Intercity	1.131		0.713–1.795	0.602	0.172	1.188	0.838–1.682	0.333
	Interprovince	0.395		0.258–0.603	0.000	0.245	1.277	0.885–1.844	0.191
Income	Lowest (<20th percentile) (Reference)								
	Lower (20–39th percentile)	1.169		0.819–1.667	0.389	0.327	1.386	0.983–1.955	0.063
	Middle (40–59th percentile)	1.296		0.918–1.830	0.140	0.512	1.668	1.141–2.440	0.008
	Higher (60–79th percentile)	1.221		0.849–1.756	0.282	0.408	1.504	0.993–2.279	0.055
	Highest (≥80th percentile)	1.758		1.206–2.562	0.003	0.757	2.132	1.255–3.622	0.005
Health insurance	BMISURR (Reference)								
	UEBMI	1.044		0.816–1.335	0.731	−0.439	0.645	0.480–0.861	0.004
Region	West (Reference)								
	Central	1.080		0.683–1.708	0.742	−0.809	0.445	0.321–1.618	0.000
	East	1.293		0.192–0.445	0.000	−0.305	0.737	0.498–1.092	0.128
*R* ^2^		0.164				0.107			
*N*		1,724				2,474			

Among rural older adult migrants, positive health determinants included receiving health education (OR = 1.416, 95% CI: 1.073–1.864), having a health record (OR = 1.462, 95% CI: 1.098–1.946), and contracting with a family physician (OR = 2.652, 95% CI: 1.674–4.200). Higher educational attainment—junior high school (OR = 3.270, 95% CI: 1.772–6.037), senior high school (OR = 5.295, 95% CI: 2.658–10.546), and university (OR = 5.642, 95% CI: 2.622–11.370)—was also associated with better self-rated health. Additionally, employment (OR = 3.790, 95% CI: 2.702–5.316) and higher income were linked to more favorable health outcomes. However, enrollment in UEBMI (OR = 0.645, 95% CI: 0.480–0.861) and residence in the central region (OR = 0.445, 95% CI: 0.321–0.618) were associated with poorer self-rated health.

### 4.3 Decomposition analysis

#### 4.3.1 Utilization of public health services

Table 8 presents the results of the overall Blinder–Oaxaca decomposition, analyzing the differences in public health service utilization among older adult migrants in urban and rural areas. In terms of health education, the results indicate that the unexplained component accounts for a greater proportion of the observed differences between urban and rural older adult migrants. This suggests that unobservable factors—such as systemic disparities, policy implementation differences, or cultural influences—play a significant role in enabling better access to health education for urban older adult migrants. Notably, the explained component is negative, indicating that when only observable factors are considered, rural older adult migrants would be expected to have even lower levels of health education, further highlighting the role of unmeasured variables. In terms of health record establishment, the results show that the explainable component accounts for a greater share of the differences between urban and rural older adult migrants. This implies that observable factors—such as differences in socioeconomic status, education, insurance coverage, or mobility patterns—substantially contribute to disparities in health record establishment. Similarly, in the terms of family physician contract signing, the explained component also contributes more significantly to the observed differences. This finding indicates that disparities in access to family physician services between urban and rural older adult migrants are largely attributable to measurable characteristics and structural differences.

**Table 8:** Overall Blinder–Oaxaca decomposition of differences in the utilization of public health services among the older adult migrant population.

Public health services	Component	*β*	95%CI	*p*	Contribution (%)
Health education	Explained difference	−0.030	−0.047 to −0.012	0.001	−115.38%
	Unexplained difference	0.056	0.025–0.088	0.000	215.38%
	Total difference	0.026	−0.0002 to 0.053	0.052	
Health record	Explained difference	0.043	0.024–0.061	0.000	165.38%
	Unexplained difference	−0.016	−0.048 to 0.016	0.323	−61.54%
	Total difference	0.026	−0.002 to 0.054	0.066	
Family doctor contract	Explained difference	0.042	0.029–0.056	0.000	110.53%
	Unexplained difference	−0.005	−0.029 to 0.019	0.697	−13.16%
	Total difference	0.038	0.016–0.059	0.000	

*β*, regression coefficients; CI, confidence interval.

Table 9 presents the specific Blinder–Oaxaca decomposition results of differences in public health service utilization among older adult migrants across urban and rural settings. In the domain of health education, several factors significantly contribute to the observed disparities. These include age (5.88%), educational attainment (26.47%), migration scope (13.24%), household income (8.82%), and health insurance coverage (26.47%). Among these, age, migration scope, and household income exert negative effects, suggesting that these variables may exacerbate urban–rural disparities in health education. In contrast, educational attainment and health insurance serve as positive contributors, meaning they help to reduce these disparities. With regard to health record establishment, age (3.54%), migration scope (24.79%), health insurance (16.53%), and geographic region (38.96%) are identified as significant explanatory variables. Age, migration scope, and region negatively influence urban–rural differences, thereby potentially widening the gap in health record utilization. Conversely, health insurance exerts a positive influence, indicating its potential to mitigate these disparities. In the area of family physician contract signing, age (4.00%), migration scope (28.00%), and region (48.00%) are the main explanatory factors. All three are associated with widening urban–rural disparities in access to family doctor services among the older adult migrant population.

**Table 9:** Detailed Blinder–Oaxaca decomposition of differences in utilization of public health services among the older adult migrant population.

Variables	Component	Coefficient	Health education	Health record	Family doctor contract
Age	Explained component	*β*	0.004	0.003	0.002
		95%CI	0.001–0.008	−0.0002 to 0.007	−0.0003 to 0.005
		*p*	0.025	0.066	0.082
		Contribution (%)	5.88%	3.54%	4.00%
	Unexplained component	*β*	0.041	0.042	0.028
		95%CI	−0.019 to 0.101	−0.020 to 0.104	−0.019 to 0.075
		*p*	0.185	0.184	0.249
		Contribution (%)	9.11%	16.96%	14.97%
Education	Explained component	*β*	−0.018	−0.008	0.002
		95%CI	−0.032 to −0.005	−0.021 to 0.006	−0.008 to 0.011
		*p*	0.009	0.274	0.702
		Contribution (%)	−26.47%	−9.45%	4.00%
	Unexplained component	*β*	0.002	0.005	0.030
		95%CI	−0.094 to 0.098	−0.094 to 0.104	−0.045 to 0.105
		*p*	0.969	0.922	0.426
		Contribution (%)	0.969	2.02%	16.04%
Work	Explained component	*β*	−0.001	0.0007	0.001
		95%CI	−0.002 to 0.001	−0.0008 to 0.002	−0.0004 to 0.003
		*p*	0.341	0.381	0.133
		Contribution (%)	−1.47%	0.83%	2.00%
	Unexplained component	*β*	0.029	0.026	0.044
		95%CI	−0.028 to 0.086	−0.033 to 0.846	−0.0001 to 0.089
		*p*	0.321	0.390	0.051
		Contribution (%)	6.44%	10.50%	23.53%
Mobility range	Explained component	*β*	0.009	0.021	0.014
		95%CI	0.002–0.015	0.013–0.029	0.008–0.019
		*p*	0.009	0.000	0.000
		Contribution (%)	13.24%	24.79%	28.00%
	Unexplained component	*β*	0.084	0.134	0.064
		95%CI	−0.006 to 0.174	0.041–0.226	−0.005 to 0.134
		*p*	0.067	0.005	0.071
		Contribution (%)	18.67%	54.10%	34.22%
Income	Explained component	*β*	0.006	0.005	0.003
		95%CI	−0.001 to 0.013	−0.001 to 0.012	−0.002 to 0.008
		*p*	0.055	0.110	0.203
		Contribution (%)	8.82%	5.90%	6.00%
	Unexplained component	*β*	0.014	0.028	−0.014
		95%CI	−0.043 to 0.698	−0.031 to 0.086	−0.058 to 0.031
		*p*	0.637	0.353	0.547
		Contribution (%)	3.11%	11.30%	−7.49%
Health insurance	Explained component	*β*	−0.018	−0.014	−0.004
		95%CI	−0.028 to −0.009	−0.023 to −0.005	−0.011 to 0.002
		*p*	0.000	0.003	0.214
		Contribution (%)	−26.47%	−16.53%	−8.00%
	Unexplained component	*β*	−0.189	−0.0007	0.005
		95%CI	−0.029 to 0.001	−0.015 to 0.016	−0.007 to 0.016
		*p*	0.063	0.926	0.429
		Contribution (%)	−42.00%	−0.28%	2.67%
Region	Explained component	*β*	−0.012	0.033	0.024
		95%CI	−0.026 to 0.002	0.019–0.048	0.013–0.035
		*p*	0.102	0.000	0.000
		Contribution (%)	−17.65%	38.96%	48.00%
	Unexplained component	*β*	0.091	0.012	−0.002
		95%CI	−0.269 to −0.111	−0.069–0.093	−0.064 to 0.059
		*p*	0.000	0.778	0.942
		Contribution (%)	20.22%	4.84%	−1.07%

#### 4.3.2 Health

Table 10 presents the results of the overall Blinder–Oaxaca decomposition of health disparities among older adult migrants. The findings indicate that 30.98% of the health disparities between urban and rural older adult migrants can be attributed to explained (observable) factors. In contrast, the remaining 69.02% is accounted for by unexplained (unobservable) factors.

**Table 10:** Overall Blinder–Oaxaca decomposition of health disparities among older adult migrants.

Component	*β*	95%CI	*p*	Contribution (%)
Explained difference	0.101	0.069–0.132	0.000	30.98%
Unexplained difference	0.225	0.182–0.269	0.000	69.02%
Total difference	0.326	0.288–0.364	0.000	

*β*, regression coefficients; CI, confidence interval.

Table 11 presents the results of the specific Blinder–Oaxaca decomposition of health disparities among the older adult migrant population. The analysis reveals that family doctor contracting (1.99%), age (13.45%), education (13.45%), employment status (2.49%), migration scope (15.94%), income (15.94%), and region (43.33%) are statistically significant contributors to the observed health differences between urban and rural older adult migrants. Among these, family doctor contracting, age, migration scope, and region act as negative factors, suggesting they may exacerbate health disparities. Conversely, educational attainment, employment, and income serve as positive factors, potentially mitigating health differences across urban and rural settings.

**Table 11:** Detailed Blinder–Oaxaca decomposition of health disparities among older adult migrants.

Variables	Explained component				Unexplained component			
	*β*	95%CI	*p*	Contribution (%)	*β*	95%CI	*p*	Contribution (%)
Health education	0.0004	−0.001 to 0.002	0.607	0.20%	0.027	−0.034 to 0.088	0.379	1.69%
Health record	0.0004	−0.002 to 0.003	0.679	0.20%	−0.004	−0.032 to 0.024	0.773	−0.25%
Family doctor contract	0.004	−0.0004 to 0.009	0.080	1.99%	−0.021	−0.040 to −0.001	0.036	−1.31%
Age	0.027	0.016–0.038	0.000	13.45%	0.366	0.287–0.444	0.000	22.86%
Education	−0.027	−0.047 to −0.007	0.007	−13.45%	0.168	0.044–0.293	0.008	10.49%
Work	−0.005	−0.010 to 0.0004	0.071	−2.49%	0.054	−0.019 to 0.129	0.151	3.37%
Mobility range	0.032	0.021–0.044	0.000	15.94%	0.497	0.377–0.617	0.000	31.04%
Income	−0.016	−0.026 to −0.006	0.002	−15.94%	−0.061	−0.134 to 0.012	0.098	−3.81%
Health insurance	−0.002	−0.015 to 0.010	0.705	−1.00%	−0.024	−0.043 to −0.005	0.014	−1.50%
Region	0.087	0.065–0.110	0.000	43.33%	0.379	0.274–0.485	0.000	23.67%

## 5 Discussion

This study aimed to analyze the differences in public health service utilization and health between urban and rural older adult migrants and the impact of different factors on such differences. Our findings indicate that the proportion of older adult migrants in rural areas utilizing the three types of public health services, namely health education, health records, and family doctor contracting, is slightly higher than that of urban older adult migrants. However, their self-rated health status was significantly lower than that of urban older adult migrants. These results align in part with, but also diverge from, previous studies. For example, Wu et al. reported that older adult migrants with rural hukou demonstrated greater awareness of public health service programs and were more likely to have established health records and received health education on chronic diseases than urban older adult migrants (76). Similarly, Cai et al. found that urban older adult migrants reported significantly better self-rated health than rural older adult migrants (77). In contrast, findings by Wang et al. suggested that both the acceptance of health education and the establishment of health records were lower among older adult migrants in rural areas than among those in urban areas (78). Other related studies have similarly concluded that the lower public health service utilization observed among rural older adult migrants may be attributed to the uneven distribution of healthcare resources, limited access to health-related information, and inadequate social support networks (79, 80).

This study concludes that although rural older adult migrants exhibit a higher utilization rate of public health services compared to their urban counterparts, their overall health status remains poorer. Several factors contribute to this paradox. First, regarding service accessibility, government policy initiatives have enhanced rural migrants’ access to basic public health services. In recent years, Chinese health reforms have explicitly prioritized the equalization of public health services for the floating population, aiming to gradually reduce urban–rural disparities in service provision. These policies have effectively improved service utilization among rural older adult migrants and narrowed the urban–rural gap in access to public health services.

Second, in terms of accessibility barriers, urban older adult migrants encounter fewer structural and individual-level challenges compared to their rural counterparts (81). These challenges encompass financial capacity, educational attainment, health literacy, social integration, and access to comprehensive healthcare coverage. For instance, rural older adult migrants often have lower incomes and limited financial resources, which restrict their ability to afford services necessary for maintaining good health. Moreover, lower levels of health literacy, stemming from prior unhealthy lifestyles and limited access to health information, may result in inadequate health management practices. According to Anderson’s Health Behavior Model, predisposing characteristics, enabling resources, and need factors influence both health behaviors and outcomes (82). The compounded disadvantages faced by rural older adult migrants across these dimensions may therefore hinder the effectiveness of their public health service utilization, preventing measurable improvements in health within a short timeframe. Additionally, disparities in healthcare coverage further compound the issue. Rural older adult migrants may struggle to access consistent and comprehensive healthcare services across different regions. They often lack urban health insurance or other forms of healthcare support due to their transient status, which limits their access to services such as chronic disease management and long-term care (83), thereby adversely affecting their overall health. Regarding social adaptation, the rural older adult migrant population typically comes from culturally homogeneous backgrounds, which intensifies the challenges they face in adjusting to the urban environment. Differences in language, lifestyle, and social norms often make it difficult for them to integrate into urban communities (84). Moreover, after relocating to cities, many rural older adult migrants lose their original sources of familial and community support. This disruption can lead to social isolation and a lack of daily interpersonal interaction and emotional support, which may further compromise their physical and psychological wellbeing.

In addition, in terms of service quality, many members of the migrant population belong to low-income groups and often lack the financial means to access high-quality health resources. In contrast, urban older adult migrants, who generally possess advantages in income and health insurance coverage, are better positioned to utilize higher-quality medical and public health services to maintain and improve their health. Existing studies have consistently shown that the income levels of rural migrants are significantly lower than those of their urban counterparts, and their use of healthcare services is also markedly limited (21, 85). Therefore, public health services provided by the government have become an important channel through which rural older adult migrants obtain health protection.

Finally, in terms of baseline health and disease burden, rural areas remain disadvantaged with respect to the availability of medical resources and the capacity of healthcare services (86). Health management for the older adult in these areas tends to lack detail and continuity, resulting in delayed or inadequate treatment when medical needs arise. Particularly for illnesses requiring complex interventions, the basic health infrastructure in rural regions is often insufficient, leading to unmet healthcare needs (87). Many older adult individuals already suffer from chronic conditions such as hypertension and diabetes, yet due to limited access to care, they are unable to receive timely and appropriate interventions. After migrating to urban areas, these individuals often experience interruptions in their health management due to their transient lifestyles, which further hinders improvements in their health outcomes. These findings indicate that although China’s policy of equalization of basic public health services has safeguarded the health rights and interests of the migrant population to a certain extent and contributed to the goal of achieving health for all, there is still a need to further improve this system. It is essential to strengthen the effectiveness of public health services in protecting the health of rural older adult migrants and in narrowing the gap in health equity. Notably, the conclusions of this study are based on cross-sectional data, which limits the analysis to a static snapshot of disparities at a particular point in time. Future research using longitudinal data would allow for the examination of dynamic trends and changes in the distribution of public health service utilization and health disparities over time, thereby offering a more comprehensive understanding of urban–rural differences among older adult migrants.

In analyzing the factors influencing public health service utilization and health among older adult migrants, this study extends previous research (18, 88) by incorporating two key innovations. First, it treats the three primary public health service programs—health education, health records, and family doctor contracting—as well as health status, as an integrated analytical framework. This approach not only examines the differential impact of commonly cited influencing factors on the utilization of public health services and health outcomes among older adult migrants, but also further explores the health effects associated with public health service utilization. In doing so, it clarifies the relationship between the use of public health services and the health status of this population. Second, the study investigates these relationships from an urban–rural perspective, allowing for a more precise identification of individualized factors influencing service utilization and health outcomes among urban and rural older adult migrants. This analysis contributes evidence to support targeted policy design and tailored interventions. Our findings indicate notable urban–rural differences in the determinants of both public health service utilization and health status among older adult migrants. Moreover, the analysis deepens the application of Anderson’s Health Behavior Model by elaborating on the influence of predisposing characteristics, enabling resources, and situational factors. Specifically, for urban older adult migrants, age, migration range, income, region, education level, and health insurance are the primary determinants of access to health education, whereas specifically, for urban older adult migrants, age, migration scope, income, region, education level, and health insurance are the primary determinants of access to health education. Regarding health record establishment, the key influencing factors for urban older adult migrants include education level, migration scope, region, and health insurance; these same factors also apply to the rural older adult group. In the case of family doctor contracting, age, employment, migration scope, and region are the main factors for the urban older adult, while migration scope, income, health insurance, and region are more significant for rural older adult migrants. Regarding the influencing factors of health, both in urban and rural areas, older adults who received health education, established health records, and signed a contract with a family doctor were more likely to have better health, suggesting that the utilization of public health services will help improve the health status of urban and rural older migrant populations. This finding aligns with existing research, supporting the view that China’s public health equalization policy for the migrant population yields beneficial health outcomes (89, 90). Additionally, the health of urban older adult migrants is primarily influenced by education level, employment, income, region, migration scope, and age, whereas for rural older adult migrants, the most significant factors are education level, employment, income, and health insurance Overall, the results of the multiple linear regression analysis suggest that age, migration scope, education level, income, health insurance, and region are the principal determinants of public health service utilization and health among urban and rural older adult migrants. These variables are consistent with those emphasized in previous studies (91, 92), and should therefore be prioritized in efforts to reduce disparities in service utilization and health outcomes between urban and rural older adult populations. Nevertheless, it is important to acknowledge that these conclusions are derived from cross-sectional data from the 2018 CMDS. Future studies utilizing longitudinal data would provide deeper insights into how the influence of these factors evolves over time, thereby facilitating a more robust identification of key long-term determinants of public health service utilization and health among older adult migrants.

The study further applied the Blinder–Oaxaca decomposition method to explore the contribution of different factors to public health service utilization and health disparities among urban and rural older adult migrants. Compared to existing studies, this study broadens the analytical perspective by offering a more comprehensive and detailed understanding of the factors contributing to disparities in public health service utilization and health outcomes between urban and rural older adult migrants. By emphasizing urban–rural differences, it provides richer empirical evidence to support efforts aimed at reducing these disparities. For example, Shuhui et al. limited utilization of public health services and poor health outcomes among older adult migrants were predominantly concentrated within the low-income population. The most significant contributor to inequitable utilization of public health services was per capita monthly household income, accounting for 74.354 and 53.383% of the disparities in the utilization of health records and family doctor services, respectively. This was followed by the range of mobility, which contributed 43.474% to the inequity in health record utilization and 32.063% in family doctor service use. With respect to health disparities, the leading contributing factor was also per capita monthly household income, explaining 59.561% of the inequality in self-assessed health and 66.641% in illness experienced in the previous year. Min et al. also found that disparities in self-assessed health were significantly influenced by household registration type (36.347%), while disparities in illness within the past year were primarily associated with the range of mobility (14.155%). Similarly, household registration contributed 14.154% to disparities in self-assessed health, and mobility range contributed 14.153% to disparities in illness experienced in the past year (58). In contrast, the present study adopts an urban–rural comparative perspective to further examine the contributing factors behind disparities in public health service utilization and health outcomes among older adult migrants. The findings indicate that age, mobility range, and household income are key factors contributing to differences in access to health education between urban and rural older adult migrants. Furthermore, age, mobility range, and region significantly affect disparities in the establishment of health records and family doctor contracting. With respect to health outcomes, differences in family doctor contracting, age, mobility range, and region were identified as the main contributors to urban–rural health disparities among older adult migrants. It should be noted that these findings are based on cross-sectional data from the 2018 CMDS. Therefore, future research using longitudinal data would allow for a more nuanced understanding of the evolving influence of these factors over time and support more targeted efforts to address disparities in public health service utilization and health outcomes among older adult migrant populations across urban and rural areas.

## 6 Policy recommendations

Based on our findings, we propose the following recommendations. First, the accessibility of public health services for older adult migrants should be further improved. At present, the utilization of such services among some urban and rural older adult migrants remains insufficient. In the future, equity in public health service utilization can be enhanced by expanding the scope of services covered and ensuring their availability to a broader segment of the migrant older adult population. Second, public health education targeting the older adult migrant population should be strengthened. Efforts to disseminate health knowledge and improve health literacy are essential to increase awareness and promote greater use of public health services. This will help prevent the phenomenon of “underutilization of available basic public health services” among this group. Third, the structure of public health services should be optimized to advance health equity among older adult migrants. Given the positive health outcomes associated with the use of public health services, it is necessary to improve the quality and relevance of health education. This includes tailoring content to the specific needs of older adults and enhancing the health education supply system to ensure that it is responsive and accessible.

Fourth, efforts to promote the establishment of health records among the older adult migrant population should be intensified. The ongoing improvement of health record management systems is essential to ensure the provision of regular health services. In parallel, dynamic monitoring and effective management of the health data of older adult migrants must be strengthened to better address their evolving health needs. Fifth, the family doctor contracting system for the older adult migrant population should be further enhanced. Comprehensive efforts must be made to improve the contracting and consultation mechanisms between family doctors and older adult migrants. Strengthening this system will enable family doctors to serve effectively as “gatekeepers” in safeguarding the health of this population. Finally, to achieve the goal of equalizing public health service utilization and improving health outcomes, particularly between urban and rural older adult migrants, it is crucial to address the underlying socio-economic determinants of health service equity. Institutional reforms and policy guidance across various sectors should be pursued to promote fair income distribution and equitable allocation of health resources. Specifically, the allocation of healthcare resources in rural areas should be optimized, and health insurance coverage for rural older adult migrants should be strengthened. These measures will help reduce socio-economic disparities and advance health equity between urban and rural older adult migrant populations.

## 7 Conclusion

Our regression and decomposition analyses indicate that there are differences in public health service utilization and health between urban and rural older adult migrants in China. Although the utilization of public health services among older adult migrants in rural areas is slightly higher than that among their urban counterparts, their overall health status remains lower than that of urban older adult migrants. This difference in service utilization and health between urban and rural areas is associated with various factors, including income, education, health insurance, and region. The findings have important practical implications: by providing new evidence on urban–rural differences in service utilization and health among China’s older adult migrants, these findings can inform policy optimization to further promote health among older adult migrants. To further promote equalization of public health service utilization and health equity between urban and rural older migrants, Chinese government agencies should develop targeted and region-specific intervention strategies, considering the variability of relevant factors across regions.

## 8 Strengths and weaknesses of the study

Our study provides a comprehensive description of the differences in public health service utilization and health among older adults in rural and urban areas of China, and we also explain the extent to which individual factors affecting public health service utilization and health contribute to these differences. To the best of our knowledge, this is the first study to use data from a large cross-sectional study in China to analyze differences in public service utilization and health among older migrants in urban and rural areas. This evidence from China can not only theoretically enrich the existing research on public health service utilization and the health of the older adult migrant population, but also practically support the improvement of the health protection policy for the migrant population and thus promote the realization of the dual goals of equalizing the utilization of public health services and improving the health of the migrant population. Despite its contributions, this study has several limitations. First, the analysis is based on secondary data from an open-access dataset, the most recent version of which was released in 2018. As a result, the findings may not reflect the most current trends. Future research could benefit from utilizing updated datasets to capture emerging patterns in public health service utilization and health outcomes among older adult migrants. Second, the study relies on large-scale cross-sectional data, which allows for the examination of urban–rural differences but does not permit causal inferences. Future studies should incorporate longitudinal data, such as from the Chinese General Social Survey or the China Labor Force Dynamic Survey, to assess changes over time and better evaluate the dynamic contribution of various influencing factors. Third, while the CMDS has strong national representativeness, it also has inherent limitations, such as subjective bias in respondents’ answers and uneven regional coverage. Fourth, due to the complexity of factors influencing public health service utilization and health outcomes, the study may have omitted relevant variables, including disease type, severity, and medical service use. Future research should aim to integrate these factors to provide a more comprehensive analysis. Fifth, the health and service utilization indicators used in this study were self-reported, which may introduce recall bias and subjectivity. Subsequent studies should incorporate more objective, measured indicators to enhance reliability. Sixth, the relatively small sample size resulting from the use of cross-sectional data may limit the generalizability and statistical robustness of the findings. This constraint may also reduce the ability to detect significant effects or differences. Expanding the sample size through longitudinal designs or employing complementary statistical techniques may help address these issues and improve the representativeness and scientific rigor of future studies. Finally, this study only assessed the accessibility of public health services among older adult migrants but did not examine service depth or user satisfaction—two factors that may help explain the disconnection between service utilization and health outcomes. Future research should explore these dimensions to offer deeper insights into the disparities observed between urban and rural older adult migrants.

## Data Availability

The original contributions presented in the study are included in the article/supplementary material, further inquiries can be directed to the corresponding author.
